# In Vitro and In Vivo Effects of Flavonoids on Peripheral Neuropathic Pain

**DOI:** 10.3390/molecules25051171

**Published:** 2020-03-05

**Authors:** Paramita Basu, Arpita Basu

**Affiliations:** 1 Department of Anesthesiology, Pittsburgh Center for Pain Research, University of Pittsburgh School of Medicine, Pittsburgh, PA 15213, USA; PAB171@pitt.edu; 2 Department of Kinesiology and Nutrition Sciences, School of Integrated Health Sciences, University of Nevada, Las Vegas, NV 89154, USA

**Keywords:** chemotherapy-induced peripheral neuropathy, chronic constriction injury, diabetic neuropathy, diabetic painful neuropathy, flavonoids, oxidative stress, partial sciatic nerve injury, peripheral neuropathy, spared nerve injury, spinal nerve ligation

## Abstract

Neuropathic pain is a common symptom and is associated with an impaired quality of life. It is caused by the lesion or disease of the somatosensory system. Neuropathic pain syndromes can be subdivided into two categories: central and peripheral neuropathic pain. The present review highlights the peripheral neuropathic models, including spared nerve injury, spinal nerve ligation, partial sciatic nerve injury, diabetes-induced neuropathy, chemotherapy-induced neuropathy, chronic constriction injury, and related conditions. The drugs which are currently used to attenuate peripheral neuropathy, such as antidepressants, anticonvulsants, baclofen, and clonidine, are associated with adverse side effects. These negative side effects necessitate the investigation of alternative therapeutics for treating neuropathic pain conditions. Flavonoids have been reported to alleviate neuropathic pain in murine models. The present review elucidates that several flavonoids attenuate different peripheral neuropathic pain conditions at behavioral, electrophysiological, biochemical and molecular biological levels in different murine models. Therefore, the flavonoids hold future promise and can be effectively used in treating or mitigating peripheral neuropathic conditions. Thus, future studies should focus on the structure-activity relationships among different categories of flavonoids and develop therapeutic products that enhance their antineuropathic effects.

## 1. Introduction

Pain is defined as “an unpleasant sensory and emotional experience associated with actual or potential tissue damage, as described in terms of such damage” by the International Association for the Study of Pain [1]. Pain has been classified as acute vs. chronic based on duration only, not location or cause; bone or joint, cutaneous, deep or superficial, muscle or viscera based on location; and inflammatory, neuropathic or cancer based on cause or type [2]. Pain is relayed to the spinal cord by nociceptors, which are specialized primary afferent neurons capable of detecting noxious chemical, mechanical, and thermal stimuli [3]. According to the National Center for Complementary and Integrative Health, 11 million US adults suffer with “high impact chronic pain” [4]. Neuropathic pain is one of the most intense types of chronic pain conditions, resulting from the lesion in a somatosensory nervous system, which comprises of peripheral fibers (Aβ, Aδ and C fibres) and central neurons [5]. It affects 6.9–10% of the general population as reported in a systematic review conducted by van Hecke et al. [6]. The prevalence of neuropathic pain has increased 60% in severe clinical neuropathic conditions [7]. Most of the published data on neuropathic pain conditions mainly are based on studies in participants with low back pain [8,9,10], diabetes [11], nerve entrapment syndrome [8], as well as patients who attended neurology pain centers [12]. In the UK, the data indicated the prevalence of neuropathic pain to be 8% [13]. In United States, the prevalence rates of neuropathic pain were determined as 9.8% and 12.4% based on either clinical examination or self-reporting, respectively. However, the true estimate of neuropathic pain is difficult to obtain due to differences in defining neuropathic pain as well as differences in epidemiological methods used for assessment [14].

Central and peripheral are the two main types of neuropathic pain. Central encompasses central lesion, spinal cord injury, and diseases like stroke, multiple sclerosis [5], whereas peripheral includes peripheral nerve damage or injury [15]. Numerous studies have reported the effectiveness of tricyclic antidepressants, dextromethorphan, gabapentin, lamotrigine, phenytoin, pregabalin, opioids, and tramadol for treating painful sensory neuropathy [16,17]. These therapies reduce pain by 30–50% but are accompanied by side effects, such as sedation. For gabapentinoids, the number needed to treat neuropathic pain, in most of the meta-analyses fall between 7 and 10 [18]. Furthermore, the American Academy of Neurology recommends not to use opioids and sodium valproate in neuropathic pain [19].

Despite advances in pain research by employing combinatorial chemistries and high throughput screening techniques over past 50 years, natural products like plant secondary metabolites remain important molecules of interest in the treatment of different pain conditions [20]. The need for alternative agents, such as natural products with effective and safe analgesic properties, satisfactory tolerability, and proven efficacy in treating pain conditions, has been growing throughout the world [21,22,23,24]. The present review will focus on the flavonoids, mainly anthocyanins, chalcones, flavanones, flavones, flavonols, flavan-3-ol, and isoflavones and their effects on different peripheral neuropathic pain conditions.

## 2. Flavonoids Classification, Structure, and Dietary Sources

Table 1 lists the chemical structures of different sub-groups of flavonoids. The basic structure of flavonoid includes 15 carbon atoms in their backbone, arranged in the form of C6-C3-C6 backbone with two aromatic rings (A and B) linked to each other by three carbon atoms, which may or may not give rise to the third carbon atom, which may or may not form the third ring. Flavonoids are further classified into different sub-groups, such as anthocyanins, chalcones, flavanones, flavones, flavonols, flavan-3-ol, and isoflavones based on the hydroxylation pattern and chromane ring/ring C.

Table 2 summarizes the different sub-groups of flavonoids along with their examples and dietary sources. Anthocyanins are responsible for contributing colors in flowers, fruits, vegetables, and certain grains like black rice. The color of the anthocyanin is dependent on pH in which the red is formed under acidic condition, whereas blue is formed under basic condition as well as acylation and methylation of hydroxyl groups on A and B rings [25]. The most commonly reported anthocyanins, such as cyanidin, delphinidin and pelargonidin, and their methylated derivatives constitute 90% of total anthocyanin. The hydroxylation and methylation patterns on the B ring and glycosylation with different sugar units constitute more than 500 known anthocyanidins [26,27]. Chalcones are referred to as open-chain flavonoids due to the absence of the basic C ring from the flavonoid backbone. The major chalcones are arbutin, chalconaringenin, phloretin, and phloridzin. Chalcones are present in various fruits, such as apples, berries, strawberries, pears, tomatoes, certain wheat products, and hops or beers [28,29]. In case of isoflavones, the B ring attached to the C3 position of C ring. Biochanin A, daidzein, formononetin, genistein, and glycitein, and puerarin are major isoflavones. Isoflavones are predominantly found in Leguminous plants, mainly in soybeans.

Another sub-group of flavonoids are flavanonols or dihydroflavonols, which include flavanones and their 3-hydroxy derivatives. The C ring in flavanones is saturated, which marks the difference between flavanone and flavone. The commonly reported flavanones are eriodictyol, hesperetin, and naringenin. Flavanones are generally present in citrus fruits. Taxifolin is an example of a well-known flavanonol found in citrus fruit [30,31]. Flavones, including their 3-hydrozy derivatives (flavonols), glycosides, methoxides, and other acylated products constitute the largest sub-group among all polyphenols. Flavones contain double bonds between positions 2 and 3 and a ketone in position 4 of the C ring. Flavones of the fruits and vegetables contain hydroxyl group in the 5 position of the A ring, sometimes hydroxyl group is present in 7 position of the A ring or 3′ and 4′ of the B ring, or other positions. Flavones are found as glycosides in flowers, fruits, leaves, and are also present in celery, chamomile, parsley, and red peppers. Apigenin, baicalein, isoorientin, luteolin, and tangeretin belong to flavone. Polymethoxylated flavones, such as tageretin, nobiletin and sinensetin are found in peels of the citrus fruit [32]. Flavonols contain hydroxyl group in 3 position of the C ring, which might also be glycosylated. Flavonols also possess different hydroxylation, glycosylation and methylation patterns.

Flavonols are found in teas, red wine, along with a variety of fruits and vegetables, such as apples, berries, grapes, kale, lettuce, onions, and tomatoes. The most commonly studied flavonols are fisetin, kaempferol, morin, myricetin, and quercetin. Icariin is a prenylated flavonol glycoside, which is isolated from a traditional Chinese medicinal herb *Epimedium* and is used to treat bone fractures and osteoporosis [33]. On the other hand, flavanols are also known as flavan-3-ol because a hydroxyl group is always bound at the 3 position of ring C. Unlike flavonoids, there is no double bond between C2 and C3, and no C4 carbonyl in ring C of flavonols. The presence of hydroxylation at C3 enables flavonols to have two chiral centers on the C2 and C3 molecules, hence four possible diastereoisomers. Catechin is the isomer with *trans* configuration along with two stereoisomers (+)-catechin, (−)-catechin. Epicatechin is the *cis* configuration with two stereoisomers (+)-epicatechin and (−)-epicatechin. Flavanols are found in skins of fruits like apple, bananas, blueberries, grapes, peaches and pears. Catechins, such as epicatechin, epigallocatechin, epicatechin gallate, and epigallocatechin gallate (EGCG) are found in commercially available dietary supplements, beverages, as well as whole and processed foods [34,35].

Oligomeric proanthocyanidins can be A-type or B-type structure based on the interflavanic linkages. The monomers are linked through C2–O–C7 or C2–O–C5 bonding in A-type structure, whereas the bonding involves C4–C6 or C4–C8 in B-type structure. The common procyanidin dimers and trimers include procyanidin B1, procyanidin B2, procyanidin A2, and procyanidin C1, which is a trimer. Unique dimers like theaflavin are formed when tea flavanols are fermented [36,37].

## 3. Biosynthesis of Flavonoids

Flavonoids are synthesized through the phenylpropanoid pathway by the transformation of phenylalanine into 4-coumaroyl-CoA, which enter the flavonoid biosynthesis pathway. All flavonoids are derived from the chalcone scaffold, which is biosynthesized by the enzyme chalcone synthase (CHS). The enzyme CHS catalyzes the condensation and subsequent intramolecular cyclization of three acetate units onto the p-coumaroyl-CoA, which is the end product of the general phenylpropanoid pathway. Following the enzymatic reaction catalyzed by CHS, oxoglutarate-dependent Fe^2+/3+^ dioxygenases, hydroxylases, isomerases, and reductases modify the basic flavonoid backbone and lead to the synthesis of a variety of flavonoid subclasses, such as anthocyanins, chalcones, flavanones, flavones, flavonols, and isoflavones. Chalcone isomerase stereospecifically directs the additional cyclization of chalcones to form flavones.

The acyltransferases, glycosyltransferases, and methyltransferases add acyl, sugar, and/or methyl moieties, respectively, and thus alter the solubility, reactivity, and interactions of flavonoids with different cellular targets [50,51].

## 4. Neuropathic Pain

Neuropathic pain is defined as “pain caused by a lesion or disease of the somatosensory nervous system” by the International Association for the Study of Pain [52]. Somatosensory nerves arise from fascia, joints, muscles, skin, and include various receptors, like thermoreceptors, mechanoreceptors, chemoreceptors, pruriceptors, and nociceptors that send signals to the spinal cord and eventually to the brain for further processing [5]. Neuropathic pain is difficult to treat effectively and is associated with significant impairment of health conditions and economic burden [53,54]. One of the largest studies on neuropathic pain conducted in US showed the prevalence rate of neuropathic pain being 9.8% among adults in Minnesota [55].

Neuropathic syndrome can be divided into two categories: peripheral and central neuropathic pain conditions. Central neuropathic pain is due to a lesion or disease of the spinal cord and/or brain. Cerebrovascular disease like central post-stroke pain (CPSP) affects the central somatosensory pathway as well as the neurodegenerative diseases mainly Parkinson disease cause central neuropathic pain [56]. Furthermore, spinal cord lesion or diseases like multiple sclerosis, transverse myelitis and neuromyelitis optica, spinal cord injury, and syringomyelia lead to neuropathic pain [57].

The consequences of peripheral lesion or disease lead to the development of peripheral neuropathic conditions, respectively. The pathology of peripheral disorders, which cause neuropathic pain, mainly involve unmyelinated C fibers and myelinated Aβ and Aδ fibers [58]. Some of the common and typically irreversible peripheral neuropathic conditions include alcoholic neuropathy, cancer pain, Charcot-Marie-Tooth disease, chemotherapy-induced peripheral neuropathy (CIPN), chronic inflammatory demyelinating polyneuropathy, complex regional pain syndrome, diabetic painful neuropathy (DPN), human immunodeficiency virus painful sensory neuropathy, phantom limb pain, post-herpetic neuralgia, erythromelalgia, and many more.

## 5. Chemotherapy-Induced Peripheral Neuropathy (CIPN)

The use of different chemotherapy agents and other cancer treatments will lead to the damage of the nerves away from the brain and spinal cord. CIPN is one of the most common neuropathies caused by antineoplastic agents [59]. The prevalence of CIPN is age-related, varying from 19% to more than 85% [60]. The highest incidence of CIPN has been reported in the case of platinum-based (70–100%), taxanes (11–87%), thalidomide and its analogues (20–60%), and ixabepilone (60–65%) [61]. The common symptoms for CIPN include sensitivity to cold and heat, tingling or pins-and-needles sensation, pain, burning, or numbness, difficulty with fine motor skills, and muscle weakness [62]. In general, CIPN symptoms arise after weeks or months after completion of the chemotherapy, mainly dependent on the cumulative dose of the drug [63]. Paclitaxel and oxaliplatin induce severe neuropathy during or immediately after infusion [64].

### Effects of Flavonoids on CIPN

Table 3 summarized the effects of flavonoids on CIPN. Platinum compounds play an important antitumor drug that is widely used in the treatment of various solid tumors. Cisplatin was the first synthesized drug in 1864 after carboplatin. Oxaliplatin is a third-generation platinum drug with significant cytotoxicity and diminished antitumoral resistance [65,66]. Azevedo et al. [67] reported that quercetin and rutin inhibited oxaliplatin-induced cold and mechanical nociceptive thresholds. The histopathological analysis of the skin harvested from the paws of the mice subjected to oxaliplatin-induced peripheral neuropathy showed that quercetin and rutin prevented the formation of little lacunar spaces between collagen fibers. Quercetin or rutin also prevented oxaliplatin-induced lipid peroxidation reflected by malondialdehyde (MDA) levels in the spinal cord samples. Finally, immunohistochemical analysis of the dorsal horn of the spinal cord showed that both quercetin and rutin reduced Fos expression, whereas only quercetin inhibited nitrotyrosine, and inducible nitric oxide synthase (iNOS) immunoexpression. Schwingel et al. [68] reported that quercetin nanoemulsion, and rutin reduced oxaliplatin-induced mechanical allodynia. Furthermore, the study showed that quercetin, quercetin nanoemulsion, and rutin decreased the nociceptive biomarker c-Fos in the spinal cord, indicating that phytochemicals modulated the oxaliplatin-induced neuropathy at the central level [58]. The antinociceptive activity of 6-methoxyflavone was investigated in a rat model of CIPN [69]. Thereby, 6-methoxyflavone significantly attenuated cisplatin-induced mechanical allodynia by increasing paw withdrawal threshold and thermal hypoalgesia by alleviating paw thermal threshold. Further, 6-methoxyflavone induced antinociceptive activity was without associated side effects, such as motor impairment exemplified by rotarod endurance latency [69]. In another study, naringin prevented cisplatin-induced behavioral impairment and anxiolytic-like behavior in the elevated T-maze test. Treatment of naringin significantly and dose-dependently prevented various biochemical and molecular alterations in the aged rats treated with cisplatin. Naringin prevented the increase in acetylcholinesterase activity and decrease Na^+^, K^+^-ATPase, Ca^2+^-ATPase, and Mg^2+^-ATPase activities induced by cisplatin in hippocampus. Administration of naringin with cisplatin significantly reduced oxidative biomarkers, such as lipid peroxidation, protein carbonylation, and hydrogen peroxide formation, in the hippocampus of aged rats. Naringin together with cisplatin prevented the increase in reactive oxygen species (ROS), nitric oxide (NO) level, messenger ribonucleic acid (mRNA) expression of iNOS, alteration of nonenzymatic (ascorbic acid, GSH) and antioxidant enzymes, such as superoxide dismutase (SOD), catalase (CAT), and glutathione peroxidase (GPx) activities in the hippocampus in aged rats [70]. The above study summarized the effects of naringenin in cisplatin-induced oxidative-stress-mediated inflammation in cognitive dysfunction and cognitive deficits in chemotherapy-induced peripheral neuropathy [70].

Flavonoids have also been reported in ameliorating neuropathic manifestation induced by paclitaxel [71,72,73]. Gao et al. [71] reported that quercetin significantly increased thermal hyperalgesia and mechanical allodynia in both paclitaxel treated rats and mice. In vitro treatment of quercetin on RBL-2H3 cells dose-dependently and significantly decreased the release of histamine, whereas in vivo quercetin treatment significantly reduced histamine release in the plasma of rats. Quercetin also dose-dependently decreased the protein expressions of protein kinase Cε (PKCε) and transient receptor potential V1 ion channels (TRPV1) in spinal cord and dorsal root ganglion (DRG) neurons of rats and spinal cords of mice. Furthermore, forty days of both low and high dose of quercetin treatments inhibited the translocation of PKCε from the cytoplasm to the membrane in the spinal cord and DRG of paclitaxel treated rats. Gui et al. [74] found that icariin treatment significantly alleviated paclitaxel-induced mechanical allodynia in the long term by suppressing the expressions of pro-inflammatory markers, such as tumor necrosis factor α (TNF-α), interleukin 1β (IL-1β), and interleukin 6 (IL-6), astrocytes activation, nuclear factor kappa-light-chain-enhancer of activated B cells (NF-κB) phosphorylation (p65) in the spinal cord. Icariin also reversed paclitaxel-induced downregulation of spinal sirtuin 1 (SIRT1) expression and H4 acetylation. Activation of SIRT1 and H4-K16 acetylation in the spinal cord has been reported to alleviate neuropathic pain [75]. Therefore, the study concluded that icariin suppressed paclitaxel-induced neuroinflammation and mechanical allodynia in a SIRT-1-dependent manner. Nadipelly et al. [72] reported the effects of four trimethoxy flavones in paclitaxel-induced peripheral neuropathy in mice. The flavones dose-dependently attenuated paclitaxel-induced tactile allodynia, cold allodynia and thermal hyperalgesia in mice. Flavones dose-dependently inhibited the expression of TNFα and IL-1β with IC_50_ values ranged from 60.13 µM, to 90.82 µM and 33–72 µM, respectively. Moreover, the flavones dose-dependently decreased 2,2-diphenyl-1-picrylhydrazyl (DPPH) free radicals with IC_50_ values ranging from 58.82–61.21 µM. The NO generation was also inhibited by the flavones with IC_50_ ranging from 32.39–74.59 µM [72]. Li et al. [76] reported that methoxy-substituted flavones have a better bioavailability than hydroxy flavones. Therefore, the antinociceptive activities of trimethoxy flavones against paclitaxel-induced peripheral neuropathy could be attributed to the structure-activity relationship of flavonoids, which might enhance their antinociceptive properties. In a similar study, flavonol and its dimethoxy derivatives does-dependently attenuated paclitaxel-induced tactile allodynia, cold allodynia and thermal hyperalgesia in mice along with decrease in pro-inflammatory cytokines TNFα and IL-1β and free radicals, such as DPPH and NO in a dose-dependent manner [73].

In summary, flavonoids have inhibited CIPN by attenuating pain behaviors along with inhibiting pro-inflammatory biomarkers as well as pronociceptive signaling pathways and downstream effectors. Flavonoids can be used in attenuating CIPN.

## 6. Diabetic Painful Neuropathy

Diabetic neuropathy is one of the leading causes of neuropathy. According to the International Diabetes Federation, it affects 382 million people worldwide [77]. According to an international meeting on the diagnosis and management of diabetes defined diabetic peripheral neuropathy as “the presence of symptoms and/or signs of peripheral nerve dysfunction in people with diabetes after the exclusion of other causes” [77]. Patients with diabetic neuropathy are characterized with burning, excruciating stabbing kind of pain, numbness, tingling sensation, and might also be associated with paraesthesia and hyperesthesia coupled with the aching of feet or hands [78,79].

Metabolic and vascular are the two major factors for the pathogenesis of diabetic neuropathy. Hyperglycemia leads to the accumulation of fructose and sorbitol due to increased activity of enzyme aldose reductase that confers the rate limiting step in polyol pathway. Hyperglycemia also results in the disturbance of several metabolic pathways, such as advanced glycation end products (AGE) [80,81], hexosamine [82], polyol [83,84], protein kinase C (PKC) [85,86], and poly-ADP ribose polymerase (PARP) [87,88] pathways in the nervous system. The pathways contribute in neuronal and axonal injury in diabetic neuropathy as well as increased level of oxidative stress in nervous system of diabetic neuropathy. These above-mentioned pathways also induce the production of ROS through mitochondria. The metabolic pathways and oxidative stress lead to the activation of NF-κB and specialty protein-1, resulting in neuroinflammation and vascular impairment.

### Effects of Flavonoids on Diabetic Painful Neuropathy

Table 4 reviews the effects of different flavonoids on diabetic painful neuropathy. Catechin [89], luteolin [90], pelargonidin [91], rutin [92], and genistein [93] have been reported to reduce the MDA levels in diabetic animal. MDA has been considered as a primary biomarker for free radical-mediated lipid damage and oxidative stress. The elevated level of MDA has been reported in the serum and other tissues of diabetic patients, impacting the peripheral nerve [94,95].

The flavonoids catechin [89], naringenin [96], morin [97], kaempferol [98], luteolin [90], rutin [92], genistein [93], hesperidin, and fisetin [99] have been shown to decrease the level of ROS by increase the level of different antioxidative enzymes, such as SOD, CAT, reduced glutathione peroxidase (GSH), GPx and glutathione reductase (GR) in different tissues (brain, liver, sciatic nerve) of diabetic animals. Furthermore, luteolin, morin and rutin have shown to increase the expression of nuclear factor-E2-related factor-2 (Nrf2) Nrf2 and its downstream effectors heme oxygenase-1 (HO-1) in nerve tissues of diabetic animals. Nrf-2/HO-1 plays a protective role against oxidative stress-induced damage and neuroinflammation in diabetic animals [100,101,102]. Kaempferol reduced AGEs and EGCG reduced 8-hydroxy-2-deoxyguanosine (8-OHdG), which is one of the predominant forms of free radical-induced oxidative lesions in mitochondrial and nuclear DNA [103]. Naringenin and genistein increased nerve growth factor (NGF) in sciatic nerves of diabetic animals. NGF plays important role in life maintenance and survival of the neurons. Decline in NGF induces peripheral nerve lesions like axonal atrophy, demyelination, and reduced number of nerve fibers in diabetic patients [104]. Kishore et al. [98] reported that kaempferol reduced the NO level in diabetic animals. Besides modulating different oxidative stress biomarkers, flavonoids morin, puerin and rutin reduced the expression of NF-κB, and especially morin prevented the phosphorylation of inhibitor kappa kinase (IKK) and thus prevented the translocation and expression of NF-κB. Several flavonoids decreased the levels of proinflammatory cytokines, like tumor necrosis factor-alpha (TNFα), interleukin-1beta (IL-1β), interleukin-6 (IL-6), and transforming growth factor β (TGF-β).

Matrix metalloprotease 9 (MMP-9) plays an important role in neuropathic pain and is associated with neuroinflammation and oxidative stress [105,106,107]. MMP-9 is activated by ROS produced at the site of injury. ROS inhibitor N-acetyl-cysteine suppress has been shown to suppress the expression of MMP-9 and attenuate neuropathic pain [108,109]. Catechin [89] has been shown to reduce the circulatory MMP-9 in diabetic rats.

Diabetic neuropathy in rodents have also been characterized by assessing behavioral markers, which include thermal, mechanical, chemical hyperalgesia, tactile allodynia in sensory large fibers, and sensory motors deficit in large sensory fibers [110]. The neuropathic assessment showed that flavonoids significantly downregulated chemical, mechanical, thermal hyperalgesia and allodynia in diabetic rodents. Several flavonoids, such as naringenin [96], naringin [111], luteolin [90], baicalin [112], pelargonidin [91], rutin [92], hesperidin [113], and fisetin [99], attenuated diabetes-induced thermal hyperalgesia, whereas morin [97], naringenin [96], naringin [111], kaempferol [98], luteolin [90], epigallocatechin gallate [114], and rutin [92] reduced mechanical hyperalgesia. In addition to hyperalgesia, morin [97], baicalein [112], puerarin [115], hesperidin [113], and fisetin [99] reduced mechanical allodynia, whereas, genistein [93] and naringin [111] reduced mechano-tactile allodynia, and luteolin [90] and rutin [92] reduced cold allodynia. Studies have shown that short term diabetes induced thermal, chemical and mechanical hyperalgesia [116,117], whereas long term diabetes induces thermal and mechanical hypoalgesia [118]. Baicalein ameliorated thermal hypoalgesia [119].

Electrophysiological measurements are employed in order to determine the sensory and motor nerve function, which include the assessment of motor and sensory nerve conduction in the tail and sciatic nerve. Sensory nerve conduction velocity (SNCV) is used to determine sensory nerves status in all the parameters measured in nerve conduction studies. SNCV accurately reflects the myelin integrity and axon caliber [120]. SNCV is slowed by the demyelination of the sensory nerve fibers. SNCV and motor nerve conduction velocity (MNCV) are both slow in diabetic animals. The etiology of nerve dysfunction is attributed to both vascular and non-vascular mechanisms [121]. Flavonoids, such as morin [97], rutin [92], hesperidin [113], and baicalein [119] increased both MNCV and SNCV, whereas kaempferol [98] only increased MNCV. Grape seed proanthocyanidins and its metabolites catechin and epicatechin [122] and luteolin [90] improved nerve conduction velocity in the sciatic nerve in diabetic neuropathy. Morin [97] and luteolin [90] improved nerve function by increasing nerve blood flow.

Besides attenuating oxidative stress biomarkers, pro-inflammatory cytokines, behavioral markers, and improving nerve function, flavonoid catechin increased body weight compared to diabetic animals, reduced heart hypertrophy, and reduced diabetic-induced increase in plasma glucose level [89]. Blood glucose level was also reduced by kaempferol [98], rutin [92], naringenin [96], and luteolin [90]. However, EGCG [114] and genistein [93] showed no effects on body weight and blood glucose level compared to diabetic animals. The diabetes induced body weight loss and increased plasma glucose level were ameliorated by naringenin [96]. GSPs improved diabetic parameters emphasizing mainly on LDL, which was improved when compared with diabetic control ones [122].

In conclusion, these different flavonoids can be used as the potential treatments for diabetic neuropathy. Flavonoids attenuate diabetic neuropathy by modulating different diabetic parameters, oxidative stress biomarkers, pro-inflammatory cytokines, behavioral markers, and nerve function in diabetic neuropathy.

## 7. Other Peripheral Nerve Injury Models

Majority of the neuropathic pain models use nerve injury model to induce hyperalgesia and allodynia in rodents. These models represent similarity with human neuropathic pain. Several models in rodents have developed with a mixture of intact and injured peripheral nerve fibers. Moreover, different types of nerve lesions, such as crush, complete or partial tight ligation, loose ligation with inflammatory materials and cryoneurolysis have been employed to study neuropathic pain conditions. The type of nerve lesion determines the character of the ongoing neuropathic pain. The following section of the review will focus on a few peripheral nerve injury models and the effects of flavonoids on those peripheral nerve injury models [124].

### 7.1. Sciatic Nerve Chronic Constriction Injury (CCI)

CCI is one of the most widely studied models for chronic neuropathic pain. The model was first developed by Bennett and Xie [125] and it resembles human neuropathy, resulting from trauma of peripheral nerves with nerve entrapment or compression. The model is produced by exposing the sciatic nerve on one side by making a skin incision and cutting through the connective tissues between the gluteus superficialis and biceps femoris muscles. At 1-mm intervals, four chromic gut ligatures are loosely tied around the sciatic nerves to just obstruct but not prevent the epidural blood flow. The wound is covered by placing sutures in the muscles and staples in the skin [125]. CCI leads to intraneural edema by strangulating the nerve and axotomizing nerve axons. CCI also results in thermal hyperalgesia and mechanical allodynia in mice model of CCI [126].

### 7.2. Effects of Flavonoids on Sciatic Nerve CCI Model

Table 5 summarizes the effects of flavonoids on CCI-induced neuropathic pain. CCI-induced pain includes several symptoms, including spontaneous (tingling, burning, electric-shock like) pain, dysesthesia, paraesthesia, allodynia, and hyperalgesia [127]. Flavonoids diosmin [128], hesperidin [129], and grape seed proanthocyanidins [130] reduced both thermal and mechanical hyperalgesia, whereas isoorientin [131], morin [132], EGCG [133], epigallocatechin derivate compounds 23 and 30 [134], and genistein [135] reduced only thermal hyperalgesia. Quercetin has been reported to reduce thermal and mechanical hypersensitivities in a superior degree compared to gabapentin and morphine [136]. Furthermore, pre-injury administration of quercetin showed long term effects on mechanical hypersensitivity, indicating the antinociceptive effects of quercetin in CCI model [136]. CCI-induced mechanical allodynia has been reduced by isoorientin [131], morin [132], puerarin [115], EGCG [133], and genistein [135]. Isoorientin [131] and morin [132] reduced cold allodynia. Luteolin has been reported to reduce cold and mechanical hyperalgesia, but not thermal hyperalgesia [137]. On the other hand, fisetin reduced thermal hyperalgesia, but not nociceptive sensitivity to mechanical stimuli [99].

In CCI model of neuropathic pain, oxidative/nitrosative stress and antioxidant enzymes (SOD and GSH) play important roles in the neuropathology and behavioral consequences [108,138]. Oxidative/nitrosative stress depletes the antioxidative enzymes and amplifies inflammatory processes in DRG, nerve and spinal [138,139]. The antioxidative enzymes SOD and CAT play important roles in balancing between the oxidative and prooxidative systems by scavenging free radicals and preventing peroxidative stress reaction [140]. Studies have shown that systematic injections of antioxidant reduced heat hyperalgesia [141,142]. Co-treatment of morphine with grape seed proanthocyanidins (GSPE) increased the levels of GSH, SOD, CAT, nitrate, and decreased MDA level as compared to the individual treatment with morphine or GSPE [130]. Morin restored GSH level and decreased nitrite level in the spinal cord [132] and genistein decreased the levels of ROS and MDA, increased GPx and CAT activities to combat the oxidative stress, as well as normalized nerve-injury-induced inducible nitric oxide synthase (iNOS) and neuronal nitric oxide synthase (nNOS) [135] in CCI model of neuropathic pain. Another study showed that isoorientin increased the levels of T-AOC, T-SOD, and CAT, and decreased MDA levels in CCI operated mice [131].

The increased level of nitro oxidative stress leads to the DNA damage, which in turn activates PARP, resulting in PARP-mediated DNA repair by transferring ADP-ribose units to the nuclear proteins. However, PARP activation leads to the activation of NF-κB, which in turn leads to the activation of pro-inflammatory markers, such as cyclooxygenase-2 (COX-2), iNOS, TNF-α, and IL-6, playing a major role in pain processing events [143,144]. The effects of flavonoids on different pro-inflammatory biomarkers have also been reported in CCI-induced neuropathic pain model [128]. Single treatment of diosmin reduced the mRNA expressions of IL-1β, IL-33, and St2, whereas prolonged treatment reduced the mRNA expression of TNFα along with IL-1β, IL-33, and St2. On the other hand, a single treatment reduced the expressions of microglia (Iba-1) and oligodendrocytes (Olig2) and prolonged treatment reduced astrocytes (Gfap) along with microglia and oligodendrocytes [128]. Another study showed that puerarin reduced the enhanced immunoreactivity of Iba-1 and GFAP, which are microglia and astroglia activation marker, respectively [115]. Morin reduced several inflammatory biomarkers (PARP, iNOS, COX-2, NF-κB and phospho-NF-κB, TNF-α and IL-6) in CCI-induced neuropathic pain model [132]. CCI-induced nerve injury causes the increase in DNA damage, resulting in the overactivation of PARP enzyme [145]. PARP overactivation leads to bioenergetic failure because overaction of PARP consumes a high amount of nicotinamide adenine dinucleotide (NAD) during DNA repair, and finally NAD synthesis consumes ATP, leading to the failure of ATP-dependent biochemical processes [146]. Morin treatment significantly restored CCI-induced depleted ATP levels and recused the neuronal cells from bioenergetic crisis [132]. Kuang et al. [133] reported that EGCG treatment decreased the mRNA and protein expressions of toll-like receptor (TLR4) and its endogenous ligand HMGB1. TLR4 is a pattern recognition receptor and involved in immune and inflammatory disease. Once the endogenous ligand binds to the TL4R, the receptor gets activated and induces the production of pro-inflammatory cytokines via activation of NF-κB [147,148]. Moreover, EGCG decreased downstream pro-inflammatory cytokines (IL-1β and TNF-α) of TLR4 signaling pathway, increased anti-inflammatory cytokine (IL-10), and downregulated NF-κB expression in lumbar spinal dorsal horn of CCI rats [133]. In another study EGCG and its derivate compound 30 but not compound 23 reduced mRNA and protein expressions of TNF-α, IL-1β, IL-6 in dorsal horn of spinal cord [134]. Furthermore, EGCG and compound 30 but not compound 23 reduced NF-κB protein expression in dorsal horn of spinal cord [134]. The treatment of puerarin, and isoorientin also attenuated CCI-induced pro-inflammatory cytokines (TNF-α, IL-1β, IL-6) [115,131]. Genistein also downregulated mRNA expressions of both IL-1β and IL-6 in sciatic nerve and protein expression of IL-1β in DRG and spinal cord [135].

Puerarin attenuated the overexpression of NF-κB and nuclear translocation of p65 [115], whereas genistein abolished the activation of NF-κB but did not alter its transcription in the spinal cord [135]. The expression of protein expression of NF-κB in the dorsal horn of spinal cord was attenuated by EGCG and compound 30 but not compound 23 [134].

Peripheral nerve constriction is commonly associated with loss of sensory function, mainly connected to the nerve fiber structure damage. Zhang et al. [131] reported isoorientin effectively increased the amplitude of SNCV and sensory nerve action potential (SNAP) after CCI surgery, indicating the ameliorative properties of isoorientin in CCI-induced nerve damage. Furthermore, isoorientin showed neuroprotective effects by restoring the disordered arrangement of myelinated nerve fibers, neuronal gaps, axon separation after CCI operation. Isoorientin also reduced the MMP-9 expression as well as overexpressed astrocytes and microglia in CCI operated mice [131]. MMP-9 is required for the activation of glial cell via activating pro-inflammatory cytokines [106,149]. In another study, morin treatment improved sciatic functional index (SFI) but was unable to completely recover to the normal SFI. Furthermore, morin decreased the spontaneous pain behavior and corrected foot deformity by showing no postural deficits [132].

Zhao et al. [99] explored the antinociceptive activity of fisetin, a 3,3′,4′,7-tetrahydroxyflavone, in a mouse neuropathic pain model after performing sciatic nerve CCI. The study found that fisetin exerted antihyperalgesic effect in CCI-induced animals by activating descending monoamine system with markedly increasing spinal monoamine (serotonin) levels and the ratio of 5-HT/5-HTP, and concomitantly decreasing spinal monoamine oxidase A (MAO-A). Furthermore, the study showed antihyperalgesic activity of fisetin was abolished by co-administration of 5-HT7 receptor antagonist (SB-258719), indicating the involvement of 5-HT7 in the fisetin-induced antinociceptive activity. Fisetin also attenuated the co-morbid depressive and anxiety-like behaviors developed following 2-3 weeks CCI surgery, as shown by the increased immobility time in forced swim test, increased latency to feed in novelty suppressed feeding test, and deceased lit compartment time in light-dark test [99]. In another study, the significant antihyperalgesic effect induced by the combination of hesperidin with diosmin was abolished by the presence of naloxone (opioid receptor antagonist), bicuculline (GABA_A_ receptor antagonist), and haloperidol (D2 receptor antagonist), indicating the involvement of the afore-mentioned receptors in hesperidin-diosmin-induced antihyperalgesia in CCI model of neuropathic pain [129]. In another study, co-treatment of grape seed proanthocyanidins with morphine enhanced the antihyperalgesic activity of morphine and reduced morphine-induced tolerance [130]. Luteolin has also been reported to possess antihyperalgesic effects in the spinal cord via γ-aminobutyric acid_A_ (GABA_A_) and µ-opioid receptors as evident by employing GABA_A_ and opioid receptor antagonists bicuculline and naloxone, respectively, but not via benzodiazepine and glycine receptors as evident by the application of benzodiazepine and glycine receptor antagonists, flumazenil and strychnine, respectively. Furthermore, supraspinal administration of luteolin showed no antihyperalgesic effects, indicating that this region is unlikely to contribute in antihyperalgesic effects of luteolin. Moreover, only high concentration of luteolin inhibited motor function, evident in rotarod latency experiment [137]. In another study, by employing inhibitors for NOS (L-NAME), soluble guanylate cyclase (ODQ), PKG (KT5823), and ATP sensitive potassium K^+^ channels, diosmin has been reported to inhibit CCI-induced thermal and mechanical hyperalgesia by activating NO/cGMP/PKG/KATP channel signaling pathway [128]. Valsecchi et al. [135] reported that the antinociceptive activities of genistein was mediated by ERβ because the treatment with ERβ-specific antagonist PHTPP abrogated its antinociceptive activities. The involvement of ERβ in attenuating CCI-induced neuropathic pain could be explained by the greater affinity of genistein towards ERβ than ERα [150]. The neuropathic pain is also induced by the enhancement of *N*-methyl-d-aspartate receptor (NMDAR) through the expression of NMDAR2B subunit in the dorsal horn of spinal cord [151]. Xifró et al. [134] reported the involvement of NMDAR in CCI-induced neuropathic pain in which EGCG derivative compound 30 but not EGCG and another compound 23 reduced phosphorylation and protein expression of NMDAR receptor subunit NMDAR2B in the dorsal horn of the spinal cord. Xifró et al. [134] also reported that EGCG and compound 30 but not compound 23 reduced fatty acid synthase activity (FASN) in dorsal horn of spinal cord. Although, EGCG and compounds 23 and 30 showed no effects on FASN protein expression. FASN has been well reported to play important role in neuropathic pain followed by peripheral nerve injuries [152,153,154].

### 7.3. Partial Sciatic Nerve Injury (PNI)

The partial sciatic nerve ligation model is created by ligating the dorsal third to half of the common sciatic nerve at the upper-thigh level [155]. Briefly, the left hind leg of rat is shaved and dissected to expose the sciatic nerve at the upper-thigh level. Using 8-0 silk suture, the dorsal one-third to sciatic nerve is tightly ligated just distal to the point at which thr posterior biceps semitendinosus nerve branches off [155]. A similar model developed by Malmberg and Basbaum [156] also used to study neuropathic pain and development of new therapeutics [157,158,159,160].

### 7.4. Spared Nerve Injury (SNI)

The SNI model involves selective injury of two (peroneal and tibial nerves) of the three terminal branches of the sciatic nerve, leaving the third branch (sural nerve) intact [161]. The model produced pain hypersensitivity in the territory of spared sural nerve, which is similar to the stimulus-evoked pain observed in clinical neuropathic pain syndromes [161,162].

### 7.5. Spinal Nerve Ligation (SNL)

The SNL model consists of unilateral and tight ligation of L5 and/or L6 spinal nerves of rodents at a distal location of dorsal root ganglia. The SNI model simulates human causalgia and was developed by Kim and Chung [163]. SNI model leads to increased sensitivity to heat and other non-painful mechanical stimuli [164]. Furthermore, allodynia and hyperalgesia develop quick after ligation and persist for at least four months exhibiting spontaneous pain behaviors, like guarding, licking, and lifting of the ipsilateral hind paw, but without autotomy behavior The ligation of L5/L6 spinal nerve ligation was developed by Kiso et al. and is useful in studying neuropathic pain [165]. The mechanical allodynia begins at day 1 and lasts for two months post-surgery in the L5/L6 spinal nerve ligation model.

### 7.6. Effects of Flavonoids on Other Peripheral Neuropathic Pain Models

Table 6 summarizes the effect of flavonoids on other peripheral neuropathic pain models. SNL-induced mechanical allodynia has been reduced by baicalein [166], EGCG [167], and myricetin [168]. Baicalein [166] and myricetin [168] attenuated SNL-induced thermal hyperalgesia, whereas quercetin attenuated both thermal and cold hyperalgesia in SNL rats [169]. Moreover, pre-administration of quercetin attenuated neuropathic symptoms [169]. In addition to inhibiting neuropathic pain behavior in SNL rats, quercetin inhibited inflammatory response by inhibiting pro-inflammatory cytokines TNF-α and IL-1β and reduced CCL-2 and MMPs [169], which play important roles in the pathogenesis of neuropathic pain [105,170]. The study further confirmed that quercetin inhibited TLR signaling pathway and NF-κB through activating transforming growth factor-β-activated kinase (TAK-1) [169]. Baicalein has been reported to reverse global histone-H3 acetylation and suppress HDAC1 expression [166]. Histone acetylation is catalyzed by histone acetyltransferases and removed by histone deacetylases (HDACs) [171]. Studies have shown that pharmacological intervention on the process of histone acetylation can affect pain behavior. Systematic and intrathecal administration of HDAC inhibitors provide analgesic effects in inflammatory pain models, indicating that inhibition of histone acetylation might be useful in pain management [172,173]. EGCG has been reported to completely block the neuronal NOS (nNOS) expression, but it failed to inhibit iNOS expression in SNL-induced animals [167]. Both nNOS and iNOS play important roles in mechanical hypersensitivity after peripheral nerve injury and NOS inhibitors reverse or reduce mechanical allodynia and thermal hyperalgesia in neuropathic pain [160,174]. Neuronal NOS (nNOS) is predominantly found in central nervous system and is constitutively expressed and activated by calcium signals and iNOS is activated by the pro-inflammatory cytokines and is calcium-independent [175,176,177]. Hagenacker et al. [168] reported that myricetin at low concentrations reduced voltage activated calcium channel currents (ICa_(V)_). The reduction was abolished by blocking with a PKC inhibitor chelerythrine but not by a p38 inhibitor SB203580. In contrast, high concentrations of myricetin increased voltage activated calcium channel currents (ICa_(V)_) in vitro mediated by p38 but not by PKC.

Besides SNL, flavonoids quercetin and hesperetin have been reported to reduce SNI and partial sciatic nerve ligation-induced neuropathic pain [178,179]. Muto et al. [179] evaluated the effect of quercetin on SNI model in which administration of quercetin before surgery attenuated mechanical allodynia but post-administration did not show any effect on the SNI-induced pain. Furthermore, quercetin treatment inhibited glial fibrillary acidic protein (GFAP) in satellite glial cells of ipsilateral L5 DRG. Aswar et al. [178] reported that hesperetin attenuated partial sciatic nerve ligation-induced mechanical and thermal hyperalgesia and mechanical allodynia along with downregulation of TNFα mRNA expression in sciatic nerve and different tissue biomarkers, such as total protein, NO, lipid peroxidase, and interleukins IL-1β and IL-6.

## 8. Future Directions

The present review evaluates the antinociceptive effects of flavonoids on different neuropathic pain conditions. The structurally effective flavonoids consist of catechol substructure on either A- or B- ring, together with the presence of C3 OH group of C-ring along with an oxo group on C4. The protective effects of flavonoids are enhanced by a double bond in between C2 and C3 due to the formation of a planar molecule or increase in the double bond conjugation in the flavonoids [180]. A study on structure-antioxidant activity relationships reported the protective effects of flavones, flavanones, and flavanols isolated from the resinous exudate of *Heliotropium sinuratum* due to the presence of 3-OH group [181]. The presence of 5-OH together with 4-oxo group contributed to the activity but only in presence of 3-oxo group. However, without 3-oxo group, the 5-OH with 4-oxo group is inactive.

The present review also focuses on the effects of flavonoids in combating oxidative stress either by increasing the levels of antioxidant enzymes or by decreasing the levels of ROS or other oxidative stress biomarkers in different peripheral neuropathic conditions [72,73,89,91,92,93,96,97,98,99,111,114,130,131,132,135]. Furthermore, studies have demonstrated that the antioxidant properties of flavonoids could be attributed to the increase in phenolic hydroxyls, replacement of C-3′,4′ positions in the B ring of flavonoids by hydroxyls, and the presence of a methoxyl group at the ortho position of phenolic hydroxyl [182]. Besides flavonoids, the structure-activity relationships of phytoestrogens and other phytochemicals are also known to enhance the cytotoxic and antiproliferative activities of breast carcinomas [183,184].

Therefore, the antioxidant as well as antineuropathic properties of flavonoids against different peripheral neuropathic pain conditions could be enhanced by modulating the structure-function relationships, mainly by replacing or adding different functional groups to the core flavonoid structures [180]. In addition to structure-activity relationships, future preclinical as well as clinical studies must also explore other mechanisms that may be implicated in the flavonoid-mediated amelioration of different peripheral neuropathic conditions. Future research studies must address the role of flavonoids in models of diabetes and its complications, as well as opioid use and chronic pain, which are of huge public health and economic concern globally.

## 9. Overall Conclusions

**Figure 1:**
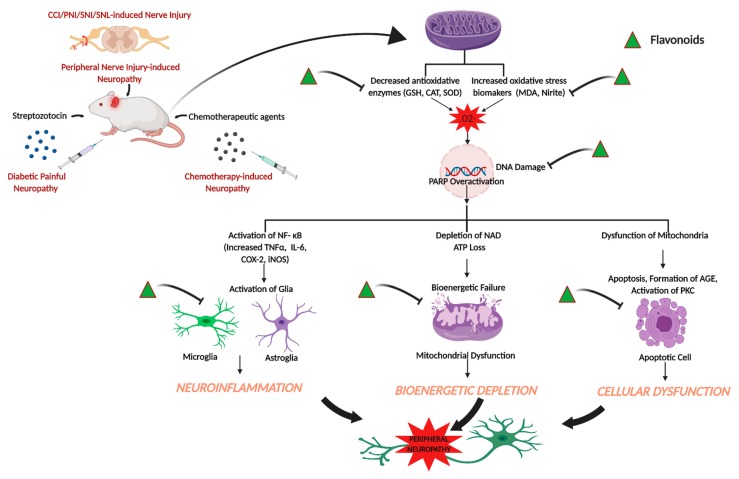
Effects of flavonoids on peripheral neuropathic conditions. Flavonoids attenuate different peripheral neuropathic pain conditions by inhibiting or downregulating different neuroinflammatory, cellular, bioenergetic and oxidative stress markers. The illustration was created with BioRender (http://BioRender.com).

The use of conventional analgesics, such as non-steroidal anti-inflammatory drugs, opioids, tricyclic antidepressants, and anti-convulsants are reported to exhibit a wide spectrum of adverse side effects, which limit the use of these drugs in treating neuropathic pain conditions. Hence, the search for alternative therapeutics with less side effects to attenuate peripheral neuropathic pain has led the researchers to identify and synthesize drugs from natural sources. The present review sheds light on the effects of flavonoids in attenuating peripheral neuropathic pain conditions. The data reported in these studies support that flavonoids are effective in attenuating neuropathic pain in different models, such as spared nerve injury, spinal nerve ligation, partial sciatic nerve injury, diabetes-induced neuropathy, chemotherapy-induced neuropathy, CCI, and related models at behavioral, biochemical, electrophysiological, histopathological, and molecular levels. Figure 1 summarizes the effects of flavonoids on peripheral neuropathic conditions based on the studies reported in the present review (Figure 1).

In conclusion, the present review provides important insights into the effects of flavonoids in alleviating different peripheral neuropathic pain conditions and how each of the flavonoids modulate different pain biomarkers in rodent models of peripheral neuropathy. Thus, there is much potential for the development of flavonoid-based potent neuropathic pain-relieving drugs in future.

## Figures and Tables

**Table 1:** Chemical structures of different sub-groups of flavonoids.

Anthocyanins		
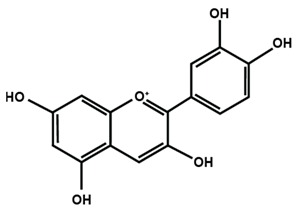	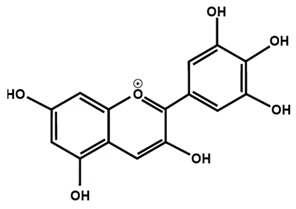	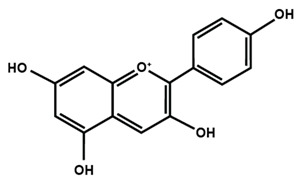
Cyanidin	Delphinidin	Pelargonidin
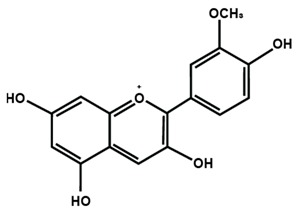	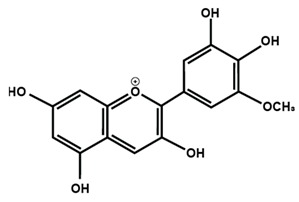	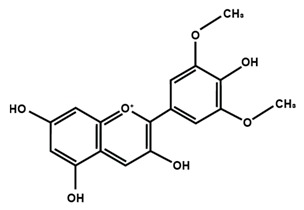
Peonidin	Petunidin	Malvidin
Chalcones		
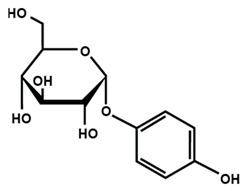	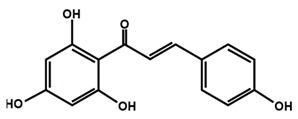	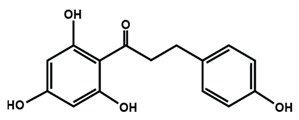
Arbutin	Chalconaringenin	Phloretin
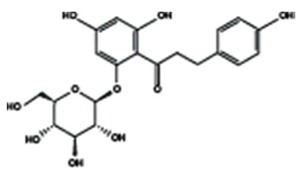	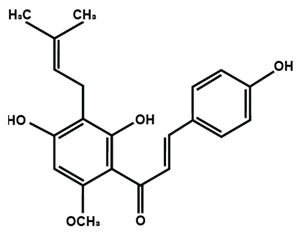	
Phloridzin	Xanthohumol	
Flavanones		
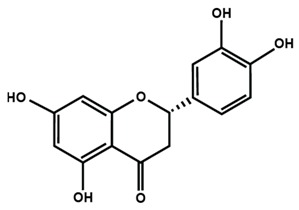	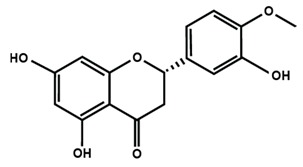	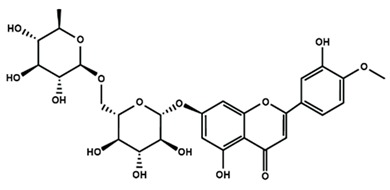
Eriodictyol	Hesperetin	Hesperidin
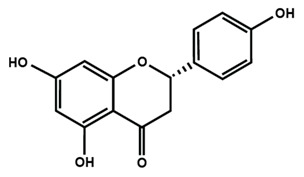	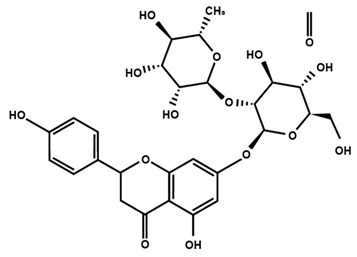	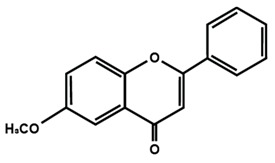
Naringenin	Naringin	6-methoxyflavanone
Flavones		
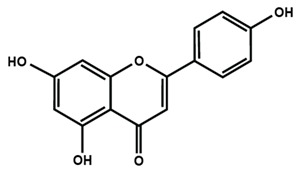	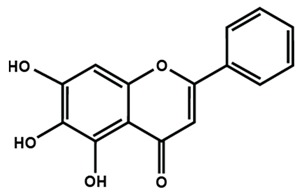	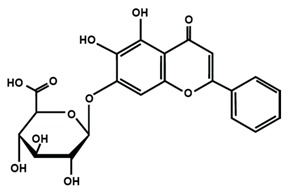
Apigenin	Baicalein	Baicalin
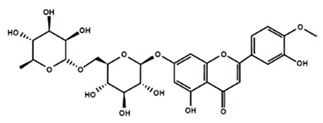	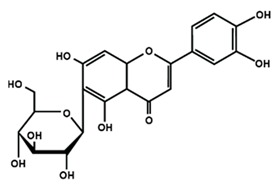	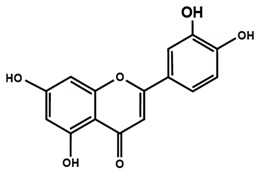
Diosmin	Isoorientin	Luteolin
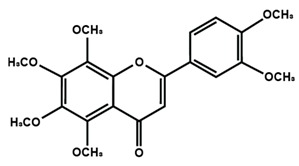	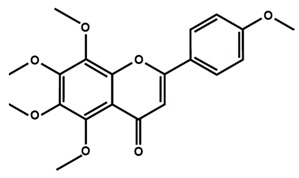	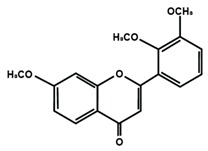
Nobiletin	Tangeretin	7,2′,3′-trimethoxy flavone
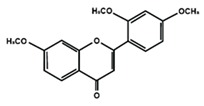	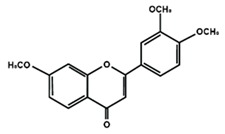	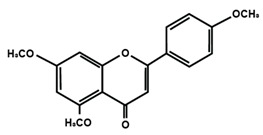
7,2′,4′-trimethoxy flavone	7,3′,4′-trimethoxy flavone	7,5,4′-trimethoxy flavone
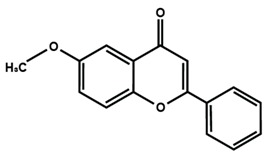		
6-methoxyflavone		
Flavonols		
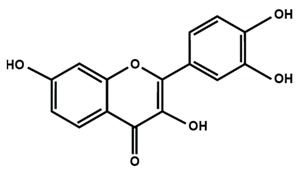	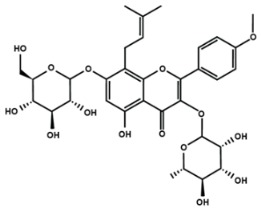	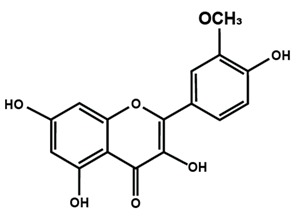
Fisetin	Icariin	Isorhamnetin
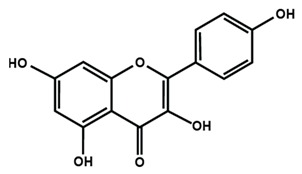	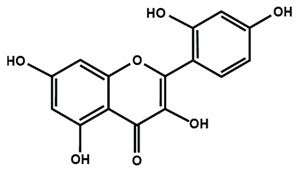	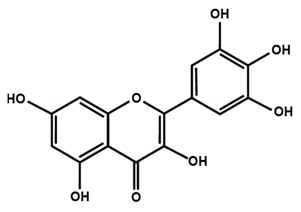
Kaempferol	Morin	Myricetin
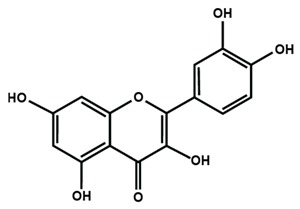	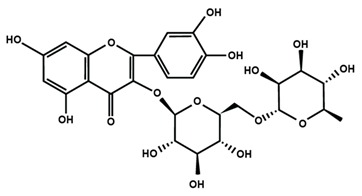	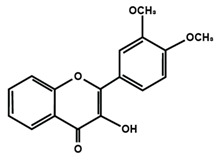
Quercetin	Rutin	3′,4′-dimethoxy flavonol
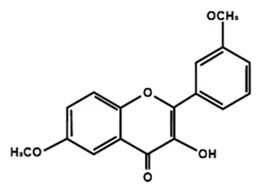	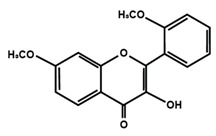	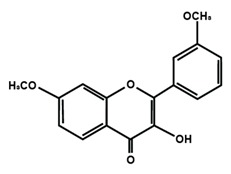
6,3′-dimethoxy flavonol	7,2′-dimethoxy flavonol	7,3′-dimethoxy flavonol
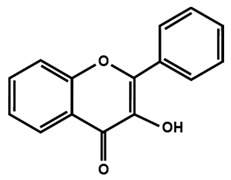		
Flavonol		
Flavan-3-ol		
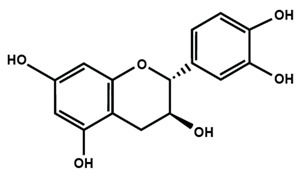	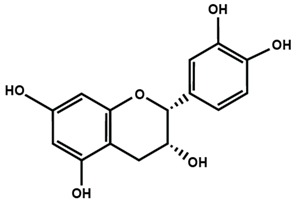	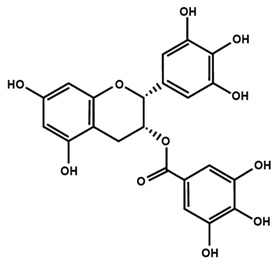
Catechin	(−)-epicatechin	Epigallocatechin gallate
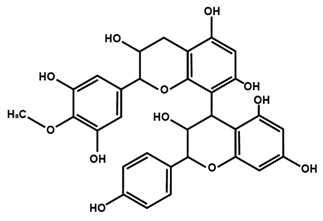		
Proanthocyanidins		
Isoflavones		
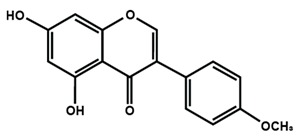	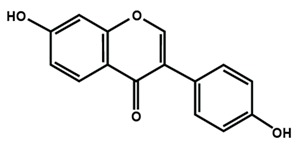	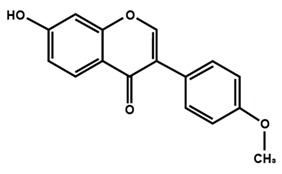
Biochanin A	Daidzein	Formononetin
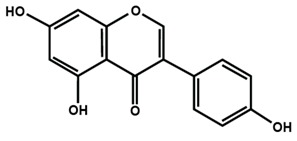	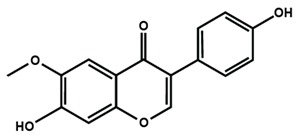	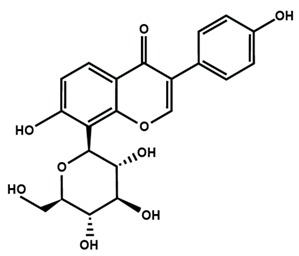
Genistein	Glycitein	Puerarin

**Table 2:** Different sub-groups, examples, and dietary sources of flavonoids.

Flavonoid Subgroup	Example	Dietary Sources	References
Anthocyanins	Cyanidin	Edible red (red clover, red hibiscus, red pineapple sage, pink blossom); blue (blue chicory, blue rosemary, cornflower); purple (purple mint, purple passionflower, purple sage); berries, blackcurrants, black carrot, purple potato, red cabbage	Khoo et al. [25]
	Delphinidin		
	Pelargonidin		
	Peonidin		
	Petunidin		
	Malvidin		
Chalcones	Arbutin	Hops, hop containing beers and herbal teas	Stevens et al. [38]; Zhao et al. [29]
	Chalconaringenin		
	Phloretin		
	Phloridzin		
	Xanthohumol		
Flavanones	Eriodictyol	Citrus fruits like lemon, lime mandarin, orange; grapefruit, herbal tea (Honeybush tea), potato	Tomás-Barberán & Clifford [39]
	Hesperetin		
	Hesperidin		
	Naringenin		
Flavones	Apigenin	Dry herbs and teas (Roman Chamomile flowers, tansy leaf, fenugreek seed, rosemary, sage, black tea, green tea, oolong tea); juices and wines (bergamot juice, mandarin orange juice, citron juice, orange juice); fruits, vegetables, olive oil and honey (kiwi, spinach, parsley, celery, lettuce, artichoke, broccoli, watermelon, pumpkin, peas); cereals and legumes (chickpea, fava pea, field pea, wheat grain, black, brown, red and white rice)	Engelhardt et al., 1993 [40]; Carnat et al., 2004 [41]; Caristi et al., 2006 [42]; Wojdylo et al., 2007 [43]; Wijaya & Mares, 2012 [44]; Pereira-Caro et al., 2013 [45]; Magalhães et al. [46]
	Baicalein		
	Diosmin		
	Isoorientin		
	Luteolin		
	Nobiletin		
	Tangeretin		
Flavonols	Fisetin	Fruits (apples, berries, grapes), vegetables (curly kale, leek, lettuce, onions, tomatoes), tea, red wine	Egert & Rimbach [47]
	Isorhamnetin		
	Kaempferol		
	Morin		
	Myricetin		
	Quercetin		
Flavan-3-ol	Catechin	Dietary supplements, beverages, whole and processed foods	Prior et al. [34]; Si et al. [35]
	(−)-Epicatechin		
	Epigallocatechin gallate		
Isoflavones	Biochanin A	Kidney beans, lentils, mung bean sprouts, mung beans, red clover, soy products, soybeans, soy products	Ho et al. [48]; Zaheer and Akhtar [49]
	Daidzein		
	Formononetin		
	Genistein		
	Glycitein		
	Puerarin		

**Table 3:** Effects of flavonoids on chemotherapy-induced peripheral neuropathy (CIPN).

Flavonoids	Animals	Dose mg/kg (Route of Administration)	Effects/Mechanisms of Action		Reference
			Behavioral Evaluation	Biochemical/Molecular Parameters	
Icariin	Male Sprague Dawley rats	25, 50, and 100 mg/kg	↓ Paclitaxel-induced mechanical allodynia in long term	↓ Paclitaxel-induced increase of TNF-α, IL-1β, and IL-6, astrocytes, NF-κB (p65) phosphorylation in spinal cord	Gui et al. [74]
		Intrathecal		Reversed paclitaxel-induced downregulation of SIRT1 expression and H4 acetylation	
7,2′,3′-trimethoxy flavone, 7,2′,4′-trimethoxy flavone, 7,3′,4′-trimethoxy flavone and 7,5,4′-trimethoxy flavone	Swiss albino mice Either sex	25, 50, 100 and 200 mg/kg	↓ Paclitaxel-induced tactile allodynia, cold allodynia and thermal hyperalgesia	X Proinflammatory cytokines (TNFα, IL-1β) and free radicals (DPPH, NO)	Nadipelly et al. [72]
		Subcutaneous			
		20, 30, 60, 120, 240 µM—in vitro			
Flavonol, 3′,4′-dimethoxy flavonol, 6,3′-dimethoxy flavonol, 7,2′-dimethoxy flavonol and 7,3′-dimethoxy flavonol	Male Swiss albino mice	25,50, 100, and 200 mg/kg	↓ Tactile allodynia, cold allodynia and thermal hyperalgesia	X TNFα, IL-1β, DPPH, NO	Sayeli et al. [73]
		Subcutaneous			
		20, 30, 60, 120, 240 µM—in vitro			
6-methoxyflavone	Male Sprague-Dawley rats	25, 50 and 75 mg/kg	↓ Cisplatin-induced mechanical allodynia and heat hypoalgesia	-	Shahid et al. [69]
		Intraperitoneal	Elicited no detectable deficit in motor control		
Quercetin	Male Sprague-Dawley rats and mice	20 and 60 mg/kg	↑ Heat hyperalgesia and mechanical allodynia in paclitaxel-treated rats and mice	↓ Expressions of PKCε and TRPV1 in spinal cords and DRGs of paclitaxel-treated rats and mice	Gao et al. [71]
		Intraperitoneal		X Translocation of PKCε from cytoplasm to membrane in spinal cord and DRG in paclitaxel-treated rats and mice	
		3, 10, 30 μM/L and 20 and 60 μM/L—in vitro		↓ Histamine release in RBL-2H3 cells in vitro as well as in plasma of quercetin-treated rats	
Naringin	Wistar rats. Sex not specified	25, 50, and 100 mg/kg	Cisplatin with naringin prevented behavioral impairment observed in only cisplatin treated group	X Cisplatin-induced increase in acetylcholinesterase	Chtourou et al. [70]
				↓ Na^+^, K^+^-ATPase, Ca^2+^-ATPase, and Mg^2+^-ATPase activities	
		Oral gavage	X Cisplatin-induced anxiogenic effect in elevated T-maze test	Altered oxidative biomarkers, antioxidant enzymes, nonenzymatic antioxidant, increase in ROS, iNOS mRNA expression, and NO levels in hippocampus	
Quercetin, quercetin nanoemulsion, and rutin	Male BALB/c mice	Quercetin, quercetin nanoemulsion, and rutin (20 mg/kg)	↓ Oxaliplatin-induced mechanical allodynia	↓ Nociceptive biomarker c-Fos in dorsal horn of spinal cord	Schwingel et al. [68]
		Oral gavage			
Quercetin and rutin	Male Swiss mice	Rutin and quercetin (25, 50, and 100 mg/kg)	X Oxaliplatin-induced peripheral neuropathy	X Lipid peroxidation, tyrosine nitrosylation, and peroxynitrite-associated neuronal damage	Azevedo et al. [67]
		Intraperitoneal			

↑ = Increased, ↓ = Attenuated/Decreased/Reduced/Suppressed, X = Inhibited/Prevented.

**Table 4:** Effects of flavonoids on diabetic painful neuropathy (DPN).

Flavonoids	Animals	Flavonoids (Dose mg/kg and Route of Administration)	Effects/Mechanisms of Action			Reference
			Behavioral Evaluation/Other Diabetic Parameters	Electrophysiological/Functional Evaluation	Histopathological/Biochemical/Molecular Parameters	
Catechin	Male Sprague Dawley rats	25 mg/kg and 50 mg/kg	↑ Body weight compared to diabetic animals	-	Improved hemodynamic parameters (heart rate, mean atrial pressure and left ventricular systolic pressure), oxidative stress parameters (MDA, GSH, CAT, SOD)	Addepalli et al. [89]
		Intraperitoneal	↓ Heart hypertrophy, plasma glucose levels		Reversed diabetes-induced neuronal damage and reduced circulatory MMP-9	
Morin	Male Sprague-Dawley rats	50 and 100 mg/kg	↓ Mechanical hyperalgesia and mechanical allodynia	Improved measurement of MNCV, SNCV, and nerve blood flow (NBF)	↑ Mitochondrial-specific superoxide dismutase 2 (SOD2) expression in high glucose-treated N2A cells	Bachewal et al. [97]
		Oral gavage			↓ Glucose-induced ROS generation by increasing expression of Nrf2 and its downstream effectors NQO1 and HO-1 in N2A cells	
		10 and 20 µM—In vitro			↓ IKK (ser176/180) phosphorylation, levels of TNFα and IL-6	
					X Translocation and expression of NF-κB in N2A cells	
					↓ Levels of TNFα and IL-6	
Grape seed proanthocyanidins (GSPs) and its metabolites C (+)-catechin; EC, (−)-epicatechin	Male Sprague-Dawley rats	250 mg/kg	GSPs - Improved diabetic parameters, especially low-density lipoprotein level	GSPs—↑ Nerve conduction velocity (NCV) in sciatic/tibial nerves	GSPs—↑ Normal mitochondria, endoplasmic reticulum in sciatic nerves and partially improved myelin sheath morphology	Ding et al. [122]
		Oral			GSPs—↓ Free Ca^2+^ concentrations and ER stress markers (GRP78, CHOP, phospho-JNK, total JNK and cleaved caspase-12)	
		(+)-catechin; EC, (−)-epicatechin			GSPs treated cells showed similar cell viability, LDH release extent, apoptosis/necrosis cell fractions to treatment with serum treated from healthy rats	
		2.5, 5, 10 µM			C and EC—Partially ameliorated cell injury in cells treated with serum from diabetic rats	
					C and EC—X Cell injury, Ca^2+^ overload and ER stress	
Kaempferol	Male Wistar rats	5 and 10 mg/kg	↓ Blood glucose level at the end of the study (90 days)	↑ MNCV compared to diabetic control rats	↑ Levels of GSH, SOD, and thiobarbituric acid reactive substances (TBARS)	Kishore et al. [98]
			↑ Diabetes-induced thermal and mechanical hyperalgesia		↓ NO level, sciatic AGEs, TNF-α, TGF-β and IL-1β	
Baicalin	Male Sprague-Dawley rats	10, 20, and 40 µg/kg	↓ Diabetes-induced mechanical allodynia and thermal hyperalgesia	-	↓ Both mRNA and protein expressions of TRPV1 in DRG of diabetic rats	Li et al. [112]
		Intraperitoneal				
6-Methoxyflavanone	Female Sprague-Dawley rats and BALB/c mice	10 and 30 mg/kg	No acute toxicity in animals ascertained by a lack of cyanosis, ataxia, convulsions, writhing or mortality	-	Thermal antinociception was antagonized by opioid receptor antagonist naloxone and GABA antagonist pentylenetetrazole	Akbar et al. [123]
		Intraperitoneal	↓ Thermal nociception in streptozotocin-induced diabetic neuropathy model at 30- and 60-min post-treatment			
			Elicited anti-allodynic and anti-vulvodynic effects			
Rutin	Male Sprague-Dawley rats	5, 25, and 50 mg/kg	↓ Plasma glucose level	↑ MNCV and SNCV in diabetic rats	↑ Na^+^, K^+^-ATPase activities in sciatic nerves	Tian et al. [92]
					↓ Caspase-3 expression in DRG neurons	
					↓ MDA and ROS levels	
					Partially increased antioxidant enzymes SOD, GPx, glutathione-S-transferase (GST), and CAT in sciatic nerves	
					↑ H_2_S, Nrf2 and HO-1 in DRG neurons	
		Intraperitoneal	↓ Diabetes-induced mechanical hyperalgesia, thermal hyperalgesia, and cold allodynia		↓ NF-κB, IкBα, p-IкBα, IL-6 and TNF-α in DRG neurons of diabetic rats	
Naringenin	Male Wister albino	25 and 50 mg/kg/day	X Fasting blood glucose level and high dose of naringenin increased insulin level	-	↓ TNFα, IL-1β and IL-6, NO level	Al-Rejaie et al. [96]
					↓ Elevated TBARS in sciatic nerves	
					↑ GSH, SOD, CAT, GPx and GR levels in sciatic nerves	
					Improved decreased sciatic expressions of insulin growth factor and NGF levels in sciatic nerves	
		Intraperitoneal	Improved mechanical and thermal hyperalgesia by increasing tail and paw withdrawal latency time		In histological analyses, low dose—partial focal peripheral axonal loss and regenerating thin myelinated axons, indicative of mild degenerative and regenerative neuropathy high dose—minimal axonal degenerative changes without regenerative features, indicative of minor degenerative neuropathy	
Luteolin	Male Sprague-Dawley rats	50 mg/kg, 100 mg/kg and 200 mg/kg	↓ Plasma glucose levels	Improved nerve function by increasing nerve blood flow (NBF) and nerve conduction velocity (NCV)	↓ ROS and MDA levels	Li et al. [90]
		Intraperitoneal	↓ Diabetes-induced cold allodynia and mechanical and thermal hyperalgesia		↑ Antioxidant enzymes SOD, GST, GPx and CAT along with Nrf2 and HO-1 in nerve tissues in diabetic rats	
Epigallocatechin-gallate (EGCG)	Male Wistar rats	2 g/L	Did not affect blood glucose concentration, body weight or liquid intake compared to diabetic animals	-	X Increase of (8-hydroxy-2-deoxyguanosine (8-OHdG) immunoreaction and Fos expression in spinal cord	Raposo et al. [114]
		Oral gavage	Ameliorated diabetes-induced tactile allodynia and mechanical hyperalgesia		X Higher percentage of 8-OHdG-IR cells that co-localized with Fos	
Fisetin	Male C57BL/6J mice	10 mg/kg	↑ Body weight and slightly decreased food/water intake	-	↓ Exacerbated oxidative stress by reducing lipid peroxide, ROS production	Zhao et al. [99]
					↑ Increased CAT activity in spinal cord, DRG, and sciatic nerve	
					Co-administration of ROS donor tert-butyl hydroperoxide(t-BOOH) abrogated antinociceptive activity	
					Co-administration of ROS scavenger phenyl-*N*-tert-butylnitrone potentiated antinociceptive activity	
		Oral gavage	Ameliorated diabetes-induced thermal hyperalgesia and mechanical allodynia		Intrathecal administration of GABA_A_ receptor antagonist bicuculline attenuated antinociceptive activity although or GABA_B_ receptor antagonist phaclofen did not alter antinociceptive activity	
Puerarin	Male Sprague-Dawley rats	4, 20, and 100 nM	Did not affect mechanical withdrawal threshold	-	↓ NF-κB, IL-6, IL-1β, and TNF-α in spinal cord	Liu et al. [115]
					X Activation of microglia and astroglia in spinal cord	
		Intrathecal	↓ Diabetes-induced mechanical allodynia		↓ Diabetes-induced elevation of TNF-α, IL-1β, and IL-6 and NF-κB DNA binding activities	
					X Overexpression of NF-κB p65 and p65 nucleus translocation	
Hesperidin	Sprague Dawley rats	25, 50 and 100 mg/kg	X Body weight loss, increased plasma glucose level, elevated intake of food and water and urine output	↑ MNCV and SNCV compared to diabetic rats	↓ Serum glucosuria, cholesterol, and triglyceride levels	Visnagri et al. [113]
	Sex not specified	Oral gavage			↓ Elevated glycated hemoglobin and aldose reductase levels, hemodynamic parameters (SBP, DBP, and MABP, neural lipid peroxidase, NO, and total calcium levels)	
					↑ Serum insulin, neural SOD, glutathione, and Na^+^K^+^ATPase levels	
			↑ Plasma glucose level compared to diabetic rats		↓ mRNA expressions of TNF-α and IL-1β	
			↑ Diabetes-induced mechano-tactile allodynia and thermal hyperalgesia		Restored diabetes-induced histological aberrations by reducing infiltration of neutrophil and macrophages	
Naringin	Male Wistar rats	40 and 80 mg/kg	Ameliorated decreased body weights and increased plasma glucose level	↑ MNCV	↑ SOD level	Kandhare et al. [111]
			↓ Diabetes-induced increase in food intake, water intake, and urine output			
		Intraperitoneal	↓ Decrease in diabetes-induced mechano-tactile allodynia, mechanical hyperalgesia, and thermal hyperalgesia		↓ TNFα, lipid peroxide, elevated neural nitrite, Na-K-ATPase levels along with percentage of apoptosis	
Baicalein	C57Bl6/J mice	30 mg/kg	↓ Weight gain	Alleviated MNCV and SNCV deficits in diabetic mice	↓ Diabetes-associated nitrated protein accumulation in sciatic nerve and normalized this variable in spinal cord	Stavniichuk et al. [119]
			Did not affect non-fasting glycemia		↓ 12(S) hydroxyeicosatetraenoic acid concentrations but did not alter sciatic nerve and spinal cord LO overexpression	
	Sex not specified	Intraperitoneal	Ameliorated thermal hypoalgesia and tactile allodynia in diabetic mice		Normalized sciatic nerve phosphorylated p38 MAPK expression without affecting total p38 MAPK expression	
Genistein	Male C57BL/6J mice	3 and 6 mg/kg	Did not alter blood glucose concentrations or body weight decrease or decrease hyperglycemia	-	↓ Pro-inflammatory cytokines TNFα, IL-1β and IL-6, ROS levels in sciatic nerves; MDA and ROS levels in brain and liver; iNOS in thoracic aorta	Valsecchi et al. [93]
					↑ NGF, eNOS and SOD	
					Did not modify decreased cerebral activities of CAT and GPx	
					Restored hepatic GPx activity but it did not modify CAT activity decrease	
		Subcutaneous	↑ Diabetes-induced mechanical allodynia		Restored the GSH content and the GSH and GSSG ratio in liver but did not modify total glutathione content	
Pelargonidin	Male Albino Wistar rats	10 mg/kg	Administration for 8 weeks prevented weight loss and reduced serum glucose level	-	↓ Increased MDA content and nitrite levels	Mirshekar et al. [91]
			↓ Serum glucose level		↑ Increased SOD level	
		Oral gavage	Ameliorated thermal hyperalgesia by increasing tail-flick response latency			

↑ = Enhanced/Increased, ↓ = Attenuated/Decreased/Reduced/Suppressed, X = Inhibited/Prevented.

**Table 5:** Effects of flavonoids on sciatic nerve chronic constriction injury (CCI).

Flavonoids	Animals	Flavonoids (Dose mg/kg and Route of Administration)	Effects/Mechanisms of Action			Reference
			Behavioral Evaluation/Other CCI-Induced Parameters	Electrophysiological/Functional Evaluation	Histopathological/Biochemical/Molecular Parameters	
Isoorientin	Male pathogen-free Institute of Cancer Research (ICR) mice	7.5, 15, and 30 mg/kg	↓ CCI-induced mechanical and cold allodynia and thermal hyperalgesia	Restored CCI-induced SNCV and SNAP	↑ Levels of total antioxidant capacity (T-AOC), total superoxide dismutase (T-SOD), CAT	Zhang et al. [131]
		Intragastrical			↓ MDA concentrations	
					X MMP-9, astrocyte, microglia overexpression in spinal cord	
					↓ Protein expressions of TNF-α, IL-6, and IL-1β in spinal cord	
					Ameliorated CCI-induced histopathological changes, such as disordered myelinated nerve fibers, swollen axons, and neuron gaps, abnormal ultrastructure of sciatic nerve and reduced abnormal myelin sheath percentage	
Diosmin	Male Swiss mice	1, 10 mg/kg	X CCI-induced mechanical and thermal hyperalgesia by NO/cGMP/PKG/KATP channel signaling pathway	-	Single treatment—X mRNA expressions of spinal cord cytokine (IL-1β, IL-33, St2)	Bertozzi et al. [128]
					Prolonged treatment—↓ TNFα mRNA expression in spinal cord	
		Intraperitoneal			Single treatment—X Glial cells activation microglia (Iba-1), oligodendrocytes (Olig2)	
					Prolonged treatment—X (Gfap), Iba-1, and Olig2 mRNA expressions	
Diosmin and Hesperidin	Male Wistar rats	Hesperidin (10, 100, 316.2, 562.3, 1000 mg/kg), Diosmin (10, 100 mg/kg)	Hesperidin—↓ Mechanical and thermal hyperalgesia	-	Combined antihyperalgesic activity mediated by D2, GABA_A_, and opioids receptors, but not 5-HT1A receptor	Carballo-Villalobos et al. [129]
		Intraperitoneal	Hesperidin + Diosmin − Improved antihyperalgesic activity			
Quercetin	Male Wistar rats	100 mg/kg	Alleviated mechanical and thermal hypersensitivity higher than morphine and gabapentin	-	-	Çivi et al. [136]
			Pre-injury administration showed long-term effects on mechanical hypersensitivity			
Grape seed Proanthocyanidins (GSPE)	Wistar rats	100 and 200 mg/kg	↓ Mechanical and thermal hyperalgesia	-	↓ MDA and nitrate levels in sciatic nerves	Kaur et al. [130]
					Co-treatment of GSPE and morphine attenuated morphine tolerance, enhanced antihyperalgesic activity	
	Either sex	Oral gavage			↑ GSH level, SOD, and CAT compared to GSPE-alone- and morphine-alone treatments	
Morin	Male Sprague-Dawley rats	15 and 30 mg/kg	Improved CCI-induced thermal hyperalgesia, mechanical and cold allodynia	Improved SFI but did not completely recover to normal SFI	Restored levels of GSH, ATP	Komirishett et al. [132]
					↓ Nitrite levels in spinal cord	
		Oral gavage	↓ Spontaneous pain, corrected foot deformity		↓ Inflammatory markers (PARP, iNOS, COX-2, NF-κB and phospho-NF-κB, TNF-α and IL-6) in spinal cord	
					↓ poly (ADP) ribose (PAR) and NF-κB levels	
EGCG and its two synthetic derivative compounds 23 and 30	Female Balb-c mice	10, 30, 50 and 100 mg/kg	EGCG and compound 30 but not compound 23—↓ CCI-induced thermal hyperalgesia	-	EGCG and compound 30 but not compound 23—↓ FASN in dorsal horn of spinal cord	Xifró et al. [134]
		Intraperitoneal			EGCG and compounds 23 and 30—No effects on FASN protein expression	
					EGCG and compound 30 but not compound 23—↓ mRNA and protein expressions of TNF-α, IL-1β, IL-6 in dorsal horn of spinal cord	
					EGCG and compound 30 but not compound 23—↓ NF-κB protein expression in dorsal horn of spinal cord	
					Compound 30 but not EGCG and compound 23—↓ Phosphorylation and protein expression of NMDAR receptor subunit NMDAR2B in dorsal horn of spinal cord	
Fisetin	Male C57BL/6J mice	5, 15 and 45 mg/kg	↓ CCI-induced thermal hyperalgesia but not nociceptive sensitivity to mechanical stimuli	-	↓ Escalated MAO-A to level like non-injured animals but did not affect MAO-B in sham or CCI mice	Zhao et al. [99]
		Oral gavage	↓ CCI-induced co-morbid depressive and anxiety-like behaviors		Exhibited antinociceptive activity with involvement of serotonergic system (coupled with 5-HT7)	
Luteolin	Male Sprague-Dawley rats	0.1–1.5 mg	Spinal administration reduced cold and mechanical, but not thermal hyperalgesia by activating GABA_A_ receptors in a flumazenil-insensitive manner and µ-opioid receptor	-	-	Hara et al. [137]
		Intrathecal or intracerebroventricular	Supraspinal administration showed no antihyperalgesic activity			
			High concentration inhibited motor function			
Puerarin	Male Sprague-Dawley rats	4, 20, and 100 nM	↓ CCI-induced mechanical allodynia	-	X Activation of microglia and astroglia in spinal dorsal horn	Liu et al. [115]
		Intrathecal			↓ TNF-α, IL-1β, IL-6, DNA binding activities, overexpression of NF-κB as well as nuclear translocation of p65	
EGCG	Male Sprague-Dawley rats	1 mg/kg	Improved CCI-induced mechanical allodynia and thermal hyperalgesia	-	↓ IL-1β and TNF-α	Kuang et al. [133]
					↑ Anti-inflammatory cytokine (IL-10)	
		Intrathecal			↓ mRNA and protein expressions of TLR4 and HMGB1	
					↓ NF-κB expression in lumbar spinal dorsal horn	
Genistein	Male C57BL/6J mice	1, 3, 7.5, 15, and 30 mg/kg	Reversed CCI-induced thermal hyperalgesia and mechanical allodynia	-	↓ mRNA expressions of both IL-1β and IL-6 in sciatic nerve and protein expression of IL-1β in DRG and spinal cord	Valsecchi et al. [135]
		Subcutaneous			↓ ROS and MDA levels	
					↑ GPX and CAT activities in CCI operated animals	
					X NF-κB activation but did not modify NF transcription in spinal cord	
					Normalized nerved injury-induced increase of iNOS and nNOS	

↑ = Enhanced/Increased, ↓ = Attenuated/Decreased/Downregulated/Reduced/Suppressed, X = Abolished/Inhibited/Prevented.

**Table 6:** Effects of flavonoids on other peripheral neuropathic pain models.

Flavonoids	Animals	Flavonoids (Dose mg/kg and Route of Administration)	Effects/Mechanisms of Action			Reference
			Behavioral Evaluation	Electrophysiological/Functional Evaluation	Biochemical/Molecular Parameters	
**SNI**						
Quercetin	Male Sprague-Dawley rats	0.1, 1%	Pre-surgery administration—↓ Mechanical allodynia	-	X GFAP in satellite glial cells of ipsilateral L5 DRG	Muto et al. [179]
		Oral gavage	Post-surgery administration—Did not affect SNI-induced pain			
**SNL**						
Quercetin	Sprague Dawley rats	10–100 mg/kg	Single or continuous administration—↓ SNL-induced thermal and cold hyperalgesia	-	↓ Phosphorylation of TAK1, IKK and JNK2 in cultured astrocytes	Ji et al. [169]
	Sex not specified	Oral gavage	Pre-surgery administration—↓ Neuropathic pain symptoms when administered		X NF- activity via TAK1 in HEK293 cells	
					↓ Protein expressions of TNF-α and IL-1β; mRNA expressions of MMP- 9, MMP-2 and CCL2	
Baicalin	Male Wistar rats	10 µg	↓ SNL-induced mechanical allodynia and thermal hyperalgesia	-	Reversed histone-H3 acetylation and HDAC1 expression of SNL-induced spinal cord dorsal horn	Cherng et al. [166]
		Intrathecal	↑Antinociceptive activity of morphine			
Epigallocatechin-3-gallate	Male Sprague Dawley rats	10–50 mg/kg	↓ SNL-induced mechanical allodynia	-	X nNOS expression in spinal cord of SNL rats	Choi et al. [167]
		Intrathecal				
Myricetin	Male Wistar rats	0.1–10 mg/kg	↓ SNL-induced mechanical allodynia and thermal hyperalgesia	Low concentrations—↓ Voltage activated calcium channel currents (I_Ca(V)_) in vitro mediated by PKC but not p38	-	Hagenacker et al. [168]
		Intraperitoneal				
		0.1–5 µM (low) 10–100 µM (high)—In vitro		High concentrations—↑ Voltage activated calcium channel currents (I_Ca(V)_) in vitro mediated by p38 but not PKC		
**Partial sciatic nerve ligation**						
Hesperetin	Wistar rats	20, 50 mg/kg	↓ Partial sciatic nerve ligation-induced mechanical and thermal hyperalgesia and mechanical allodynia	↑ Motor nerve conduction velocity	↓ TNF-α mRNA expression in sciatic nerve	Aswar et al. 2014 [178]
	Either sex	Oral gavage			↓ Different tissue biomarkers, such as total protein, NO, lipid peroxidase, IL-1β and IL-6	

↑ = Enhanced/Increased, ↓ = Attenuated/Decreased/Downregulated/Reduced/Suppressed, X = Blocked/Inhibited/Prevented.
